# Imaging the Effects of Whole-Body Vibration on the Progression of Hepatic Steatosis by Quantitative Ultrasound Based on Backscatter Envelope Statistics

**DOI:** 10.3390/pharmaceutics14040741

**Published:** 2022-03-29

**Authors:** Jui Fang, Ming-Wei Lai, Hao-Tsai Cheng, Anca Cristea, Zhuhuang Zhou, Po-Hsiang Tsui

**Affiliations:** 1 x-Dimension Center for Medical Research and Translation, China Medical University Hospital, Taichung 404332, Taiwan; juifang2014@gmail.com; 2 Division of Pediatric Gastroenterology, Department of Pediatrics, Liver Research Center, Chang Gung Memorial Hospital at Linkou, Taoyuan 333423, Taiwan; a22141@cgmh.org.tw; 3 Department of Gastroenterology and Hepatology, Chang Gung Memorial Hospital at Linkou, Taoyuan 333423, Taiwan; hautai@adm.cgmh.org.tw; 4 Department of Gastroenterology and Hepatology, New Taipei Municipal Tucheng Hospital, New Taipei City 236017, Taiwan; 5 Department of Physics and Technology, UiT the Arctic University of Norway, 9019 Tromsø, Norway; anca.cristea@npolar.no; 6 Department of Biomedical Engineering, Faculty of Environment and Life, Beijing University of Technology, Beijing 100124, China; 7 Department of Medical Imaging and Radiological Sciences, College of Medicine, Chang Gung University, Taoyuan 333323, Taiwan

**Keywords:** hepatic steatosis, ultrasound, envelope statistics, whole-body vibration

## Abstract

Hepatic steatosis causes nonalcoholic fatty liver disease. Whole-body vibration (WBV) has been recommended to allow patients who have difficulty engaging in exercise to improve the grade of hepatic steatosis. This study proposed using ultrasound parametric imaging of the homodyned K (HK) distribution to evaluate the effectiveness of WBV treatments in alleviating hepatic steatosis. Sixty mice were assigned to control (*n* = 6), sedentary (*n* = 18), WBV (*n* = 18), and exercise (swimming) (*n* = 18) groups. Mice were fed a high-fat diet to induce hepatic steatosis and underwent the intervention for 4, 8, and 16 weeks. Ultrasound scanning was performed in vivo on each mouse after the interventions for ultrasound HK imaging using the parameter *μ* (the scatterer clustering parameter). Histopathological examinations and the intraperitoneal glucose tolerance test were carried out for comparisons with ultrasound findings. At the 16th week, WBV and exercise groups demonstrated lower body weights, glucose concentrations, histopathological scores (steatosis and steatohepatitis), and *μ* parameters than the control group (*p* < 0.05). The steatosis grade was significantly lower in the WBV group (mild) than in the exercise group (moderate) (*p* < 0.05), corresponding to a reduction in the *μ* parameter. A further analysis revealed that the correlation between the steatosis grade and the *μ* parameter was 0.84 (*p* < 0.05). From this animal study we conclude that WBV may be more effective than exercise in reducing the progression of hepatic steatosis, and ultrasound HK parametric imaging is an appropriate method for evaluating WBV’s effect on hepatic steatosis.

## 1. Introduction

Hepatic steatosis, which is characterized by an increase in triglyceride-containing fat vesicles [1], may progress to nonalcoholic steatohepatitis, fibrosis, cirrhosis, and hepatocellular carcinoma [2,3]. Excess intrahepatic triglyceride is a common comorbidity of metabolic syndrome (MetS), which is generally described by several risk factors, such as elevated fasting plasma glucose, dyslipidemia, elevated blood pressure, and abdominal obesity [4]—all risk factors for type 2 diabetes mellitus and cardiovascular diseases [5,6]. Although hepatic steatosis is a hepatic manifestation of metabolic syndrome, it is unclear if hepatic steatosis is a consequence of MetS or shares the etiology of MetS [7,8]. Individuals with hepatic steatosis have presented with high blood pressure, low high-density lipoprotein cholesterol, and a constant increase in triglyceride concentrations and body mass index, which indicate the importance of including fatty liver disease into the criteria for diagnosing MetS [9].

Typically, diet control, physical activity, and exercise are major interventions for treating hepatic steatosis [10]. Although dieting may effectively reduce the grade of hepatic steatosis, severe dietary restrictions can cause muscle loss and a decline in physical fitness [11,12,13]. Maintaining a healthy diet in the long term is a challenge for most people. By contrast, physical exercise is a proven therapeutic strategy to alleviate hepatic steatosis [14]. Problems related to mechanical factors (e.g., hip and knee joints) as well as physiological factors (e.g., cardiac output and blood pressure), however, should be considered when obese or older-adult patients with hepatic steatosis exercise [15]. In this condition, swimming could be considered to reduce the risks of fatty liver disease and its associated comorbidities [16].

Recently, whole-body vibration (WBV), a newly emerging exercise alternative, received researchers’ attention and was proposed for patients who have difficulty in exercising [17]. The technique applies rapid and repeated vibrations to allow passive exercise while a patient stands or sits on a platform. Histological examinations [18,19,20] and noninvasive measurements using ultrasound FibroScan^®^ and magnetic resonance spectroscopy revealed that WBV can reduce hepatic triglyceride content and can halt the progression of hepatic steatosis [21]. Based on the above findings, WBV may be utilized as a therapeutic strategy for hepatic steatosis and needs to seek opportunities of combining with efficacy evaluation methods for integrating the diagnosis and treatment of hepatic steatosis. Essentially, WBV treatment evaluations require the involvement of noninvasive methods that are able to noninvasively characterize the liver in order to be able to routinely measure changes in the grade of hepatic steatosis following WBV treatment. In practice, noninvasive imaging compared to histological examinations is of more clinical importance for evaluating how effective WBV is in treating hepatic steatosis. Among all imaging modalities, ultrasound is recommended because it involves nonionizing radiation and is cost-effective and portable, making routine examinations and follow-up applicable.

Quantitative ultrasound can provide insights into the liver, the target of WBV treatment for hepatic steatosis. Essentially, the echoes received from liver parenchyma are formed by ultrasound backscattering because hepatocytes and lobules are smaller than the wavelength of clinically used ultrasound [22]. The locations of unresolvable scatterers in the liver could be treated as being randomly distributed, and thus ultrasound backscattered echoes generated by the interaction between the incident ultrasound and the scatterers produces random signals, which vary with changes in tissue microstructures. Considering the randomness of ultrasound backscattering, the statistical properties of the signals may be described by statistical distribution models to characterize scatterer concentrations and arrangements in a scattering medium [23]. Notably, modeling ultrasound backscattered statistics was categorized as a morphology-based ultrasound analysis approach that tends to provide insights related to histological scoring systems and benefits liver microstructure characterization [24]. Compared with other backscattered statistics models and non-statistics quantitative ultrasound techniques, the homodyned K (HK) distribution is a general backscattering model that encompasses all scattering conditions [23,25,26] and has been demonstrated to allow parametric imaging of different scattering conditions for grading hepatic steatosis [27,28]. Particularly, the HK distribution still allows characterizing tissues when the region of analysis belongs to the case of concentrated or denser media exhibiting fully developed speckle patterns in ultrasound B-scan [27]. This implies the HK parameter as a more suitable ultrasound imaging biomarker to detect moderate to severe hepatic steatosis, which tends to need WBV intervention for progress control.

In this study, we proposed using quantitative ultrasound HK parametric imaging to evaluate the effectiveness of WBV in the progress control of hepatic steatosis. Animal experiments were conducted for validating the proposed strategy. The results revealed that WBV alleviated the progression of hepatic steatosis in mice during high-fat diet feeding; HK imaging characterized WBV of the liver and correlated with the grade of hepatic steatosis obtained from histopathological examinations.

## 2. Materials and Methods

**Figure 1:**
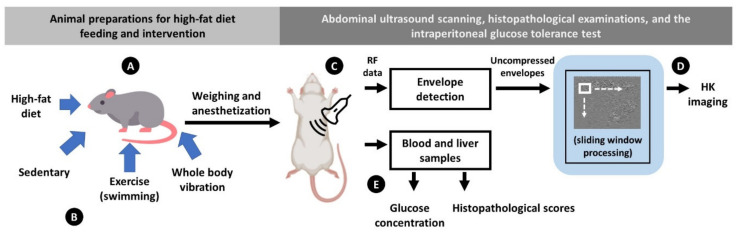
Illustration of the experimental design, including (**A**) animal preparation; (**B**) interventions; (**C**) ultrasound data analysis; (**D**) ultrasound backscattering analysis; (**E**) MetS evaluations and histopathological examinations.

The experimental design and methods are illustrated in Figure 1, including animal experiments, intervention methods, and examinations (abdominal ultrasound scanning, histopathological examinations, and the intraperitoneal glucose tolerance test). The details are described as follows.

### 2.1. Animal Preparation

The Institutional Animal Care and Use Committee of Chang Gung University approved the animal study (approval No.: CGU16-033; date of approval: 26 April 2016). Sixty 6-week-old male C57BL/6 mice were kept in standard cages with ad libitum access to food and water and habituated for a week. Six mice were randomly selected as the control (CON) group. The remaining 54 mice were randomly assigned to the sedentary (SED), WBV, and exercise groups (*n* = 18 in each group).

### 2.2. Interventions

Mice were fed a high-fat diet (HFD; 58Y1, TestDiet, Richmond, IN, USA) and were administered the intervention for 4, 8, and 16 weeks. The SED group received no intervention. Swimming was selected as an exercise intervention because mice are natural and self-motivated swimmers [29]. Mice were subjected to swimming without workload in tanks containing water at 31 ± 1 °C for 2 h a day, 5 days a week [30,31]. The WBV group were exposed to vibrations on a vertically oscillating platform (BW-760, BodyGreen, Taipei, Taiwan). During WBV, the mice were temporarily housed in one of the six compartments of an acrylic cage fixed to the top of the platform. The WBV stimulus was applied for 30 min a day, 5 days a week, at a frequency of 13 Hz, and at an acceleration of 0.68 g (1 g = 9.81 m/s^2^) and the maximum amplitude of 2 mm [32].

### 2.3. Ultrasound Data Acquisition

Each mouse was scanned in vivo after the interventions. An ultrasound imaging system (SonixTOUCH, Ultrasonix Medical, Richmond, BC, Canada) equipped with an 11-MHz linear array transducer (L14-5/38, Ultrasonix Medical, Richmond, BC, Canada) was used. The pulse length of the transducer was 0.375 mm. The mice were anesthetized with isoflurane, placed in the supine position, and had their abdomens shaved for acoustic coupling by using an ultrasound gel. The transducer was placed along the central line of the epigastric region and tilted approximately 30° against the vertical plane to identify the largest area on the left liver parenchyma for raw radiofrequency (RF) data acquisition at a sampling rate of 40 MHz. Considering the inverse square root relationship for the standard deviation of the measurement [33], five independent scans were obtained for each mouse to reduce the error by >50%.

### 2.4. Ultrasound HK Parametric Imaging

For each raw data point, an envelope image was derived from an analytic expression of each time-domain RF signal, and the corresponding B-mode image was formed using logarithm-compressed envelope images with a dynamic range of 40 dB (determined empirically to work with the SonixTOUCH system for improving the visualization of B-scan). The sliding window technique was then used for constructing HK parametric images using uncompressed envelope data. The algorithm comprises the following steps [27,28]: (i) a square window within the image is used to acquire local uncompressed envelope signals for estimating the scatterer clustering parameter (denoted by *μ* in this study) using *X* and *U* statistics [34], which are assigned as new pixels located at the center of the window. The parameter *μ* increases with the number of scatterers per resolution cell [34]. The derived parameter *k* ($k=\varepsilon /\sigma \sqrt{\mu }$), where *ε*^2^ denotes the coherent signal power and 2*σ*^2^*μ* denotes the diffuse signal power that describes the periodicity of the scatterer distribution, was not considered in the analysis because a recent study has revealed that the parameter *μ* resulted in the most suitable parameter for the task of detecting variations in scatterer properties [35]; moreover, the parameter *k* is not as effective in characterizing hepatic steatosis and fibrosis as expected [36]. The side length of the square window was five times the pulse length of the transducer, which allows a reliable construction of an HK image for the liver (working frequency range covered by the used transducers: 2–12 MHz) [27,28]. (ii) The window is moved across the range of envelope image data in distance increments with a 50% window overlap ratio. Steps (i) and (ii) are repeated to obtain a parametric mapping of *μ*. (iii) The *μ* parametric images are superimposed on the corresponding B-mode images to obtain structural and parametric information simultaneously. The algorithm for ultrasound HK imaging was programmed using MATLAB software (version R2019a, The MathWorks, Inc., Natick, MA, USA).

### 2.5. MetS Evaluations and Histopathological Examinations

MetS evaluations and histopathological examinations were performed after ultrasound scanning. The intraperitoneal glucose tolerance test was performed to assess metabolism in experimental animals [37]. The mice were fasted overnight and injected with 1 g/kg glucose into the peritoneum through saline. Blood samples were collected from the tail vein before the injection and at 30, 60, 90, and 120 min post-injection to measure glucose levels by using a handheld glucometer (ACCU-CHCK Active, Roche Diagnostics, Taipei, Taiwan). The weights of the mice were also recorded. The mice were euthanized using CO_2_ asphyxiation, and their livers were then excised. The liver samples were fixed in 10% neutral-buffered formalin, embedded in paraffin, and sliced into 4 μm thick sections for hematoxylin and eosin (H&E) staining. Histopathological examination was performed according to the following scoring system [38]. Steatosis was graded as follows: 0, none (<5% parenchymal involvement by fatty vesicles); 1, mild (5–33%); 2, moderate (33–66%); and 3, severe (>66%). Steatohepatitis was evaluated based on the sum of the grading of steatosis (0–3), lobular inflammation (0–3), and hepatocellular ballooning (0–2). Summed scores of 0–2, 3–4, and >4 indicated no steatohepatitis, a borderline case, and steatohepatitis, respectively. Liver fibrosis was evaluated using the Metavir scoring system: F0, no fibrosis; F1, portal fibrosis with no septa; F2, portal fibrosis with few septa; F3, bridging fibrosis with many septa; and F4, cirrhosis (nodular stage). Histopathological scoring was confirmed by an experienced veterinarian blinded to the experimental design.

### 2.6. Statistical Analysis

The regions of interest (ROIs) of each B-mode image were manually outlined (blood vessels and structures were excluded) by a radiologist who was experienced in animal imaging and was blinded for the analysis, and the pixel values (*μ* estimates) of the HK images corresponding to the ROIs were used for averaging. The data obtained from metabolic, histopathological, and ultrasound examinations for each mouse were provided; a one-way analysis of variance (ANOVA) was used to assess the statistical significance of the results between each intervention time point. The normality of the data was tested by the Shapiro–Wilk test. An ANOVA on ranks was applied if the normality test failed; in this condition, Dunn’s test was used for all pairwise comparisons. No fibrosis was found in any mouse (please see the section of Results), and therefore bar plots were used to describe the changes in metabolic (weight and post-injection blood glucose peak values), histopathological behaviors (steatosis, lobular inflammation, hepatocyte ballooning, and steatohepatitis scores), and the *μ* parameter as a function of the intervention time. For each time point, an ANOVA was also applied to identify the statistical significance between CON, SED, exercise, and WBV groups. The *μ* parameters were then compared with the values of weight, post-injection blood glucose, steatosis grade, lobular inflammation, hepatocyte ballooning, and steatohepatitis scores to perform a linear regression and calculate the Spearman correlation coefficient *r*. The Spearman correlation coefficient is typically used to identify the strength and direction of the monotonic relationship between the two variables measured on at least an ordinal scale; its use does not need to consider any assumptions about the distributions of the variables [39] and is relatively robust to outliers [40]. Statistical analyses were performed using SigmaPlot (version 12.0; Systat Software, Chicago, IL, USA). Significant difference was identified by *p* < 0.05.

## 3. Results

**Figure 2:**
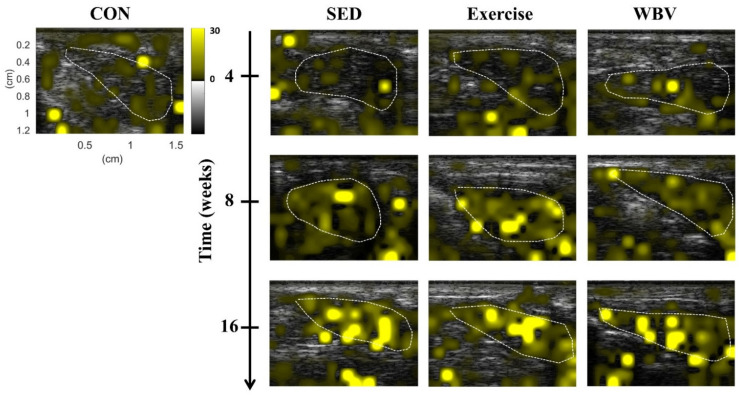
Ultrasound HK *μ* parametric images of mouse liver in various groups and intervention periods. CON: control; SED: sedentary; WBV: whole-body vibration. The delineated contours described by white dotted lines represent the ROIs for the data analysis.

**Figure 3:**
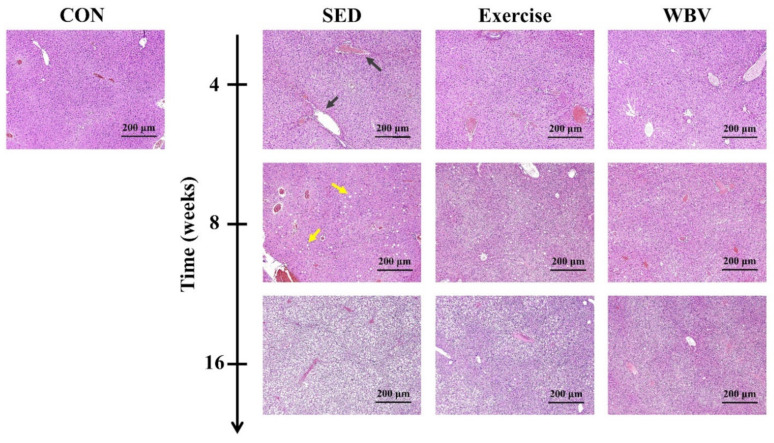
Hematoxylin-and-eosin-stained liver sections obtained from various groups and during different intervention periods. CON: control; SED: sedentary; WBV: whole-body vibration. Black arrows represent vessels; some of them contain red blood cells. Yellow arrows indicate fatty vesicles.

Figure 2 presents the representative HK *μ* parametric images of the livers in mice from the CON, SED, exercise, and WBV groups at various intervention periods. The brightness of images for all groups increased with the intervention period (i.e., HFD feeding time). However, at weeks 8 and 16, the image brightness of the WBV group increased less than those of the SED and exercise groups did. To confirm the above observations on HK imaging, the H&E-stained mouse liver sections of the CON, SED, exercise, and WBV groups corresponding to Figure 2 are shown in Figure 3. In contrast to those in the SED and exercise groups, fatty vesicles were less visible in the images of the WBV group at weeks 8 and 16. This implies that steatosis changes in the liver dominate the formation of backscattering and the corresponding HK imaging.

**Figure 4:**
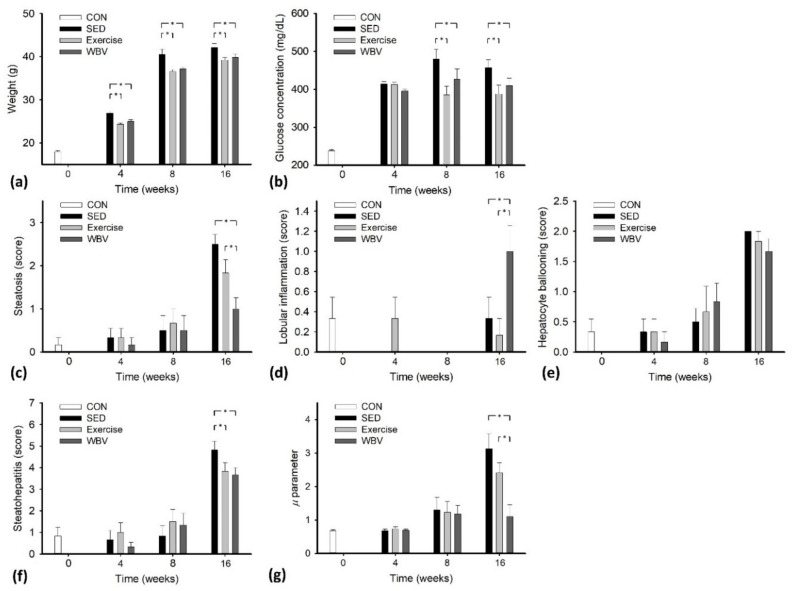
Changes in MetS, histopathological, and ultrasound examination results across the intervention periods. (**a**) Body weight, (**b**) glucose concentration, (**c**) steatosis, (**d**) lobular inflammation, (**e**) hepatocyte ballooning, (**f**) steatohepatitis, and (**g**) HK *μ* parameter (* *p* < 0.05).

Table 1, Table 2 and Table 3 show the complete experimental data. In the SED and exercise groups (Table 1 and Table 2), the HK *μ* parameter, weight, glucose concentration, steatosis grade, and scores of hepatocyte ballooning and steatohepatitis were proportional to the period of HFD feeding (*p* < 0.05). No significant difference was found in the lobular inflammation score between each intervention time point (*p* > 0.05). In the WBV group (Table 3), the weight, glucose concentration, scores of lobular inflammations, and steatohepatitis increased with feeding time (*p* < 0.05). However, for the HK *μ* parameter and steatosis grade, no significant difference existed between each time point (*p* > 0.05). This represented that WBV alleviated the progression of hepatic steatosis during HFD feeding, suppressing the increase in the HK *μ* parameter. No fibrosis was found in each group, and thus the effects of fibrosis on estimating the *μ* parameter can be excluded. Figure 4 further illustrates how the data differed between intervention periods. The body weights and glucose peak values obtained from the exercise and WBV groups were lower than those of the SED group (*p* < 0.05 for weight at weeks 4, 8, and 16; *p* < 0.05 for glucose peak value at weeks 8 and 16), as shown in Figure 4a,b. The steatosis grade and scores of hepatocyte ballooning and steatohepatitis for the exercise and WBV groups were lower than those for the SED group at week 16, as shown in Figure 4c,e,f. At week 16, the steatosis grade was significantly (*p* < 0.05) lower in the WBV group (varying around 1 (mild)) than in the exercise group (varying around 2 (moderate)), which indicated that the WBV outperformed exercise to alleviate the progression of hepatic steatosis. In this case, the *μ* parameter for the WBV group at week 16 was significantly smaller than those for the SED and exercise groups, as shown in Figure 4g, implying that HK imaging reliably reflects the efficiency of intervention. However, WBV may also have side effects because higher lobular inflammation scores were obtained in the WBV group (*p* < 0.05) than in the SED and exercise groups, as shown in Figure 4d.

**Figure 5:**
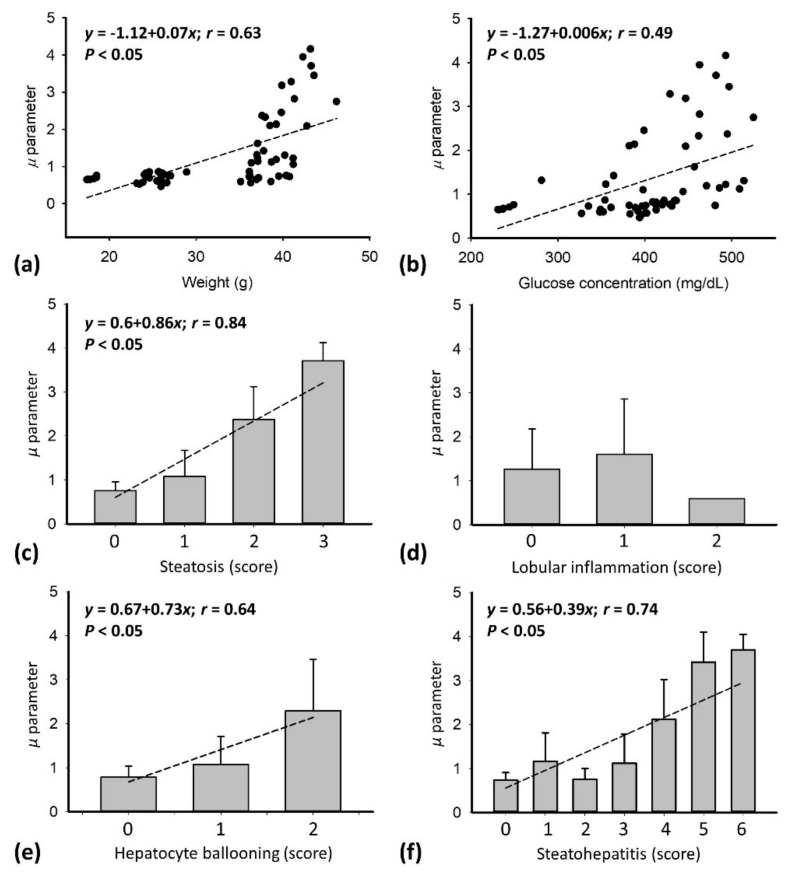
HK *μ* parameters as functions of MetS and histopathological scores (across all groups). The Spearman correlation coefficient was calculated. (**a**) Body weight, (**b**) glucose concentration, (**c**) steatosis, (**d**) lobular inflammation, (**e**) hepatocyte ballooning, and (**f**) steatohepatitis. No correlation was observed in (**d**).

Figure 5 illustrates the HK *μ* parameters as functions of MetS and histopathological scores (across all groups). The *μ* parameter was correlated with body weight (*r* = 0.63; *p* < 0.05) and glucose peak concentration (*r* = 0.49; *p* < 0.05). Furthermore, the *μ* parameter also increased with steatosis grade (*r* = 0.84; *p* < 0.05), ballooning level (*r* = 0.64; *p* < 0.05), and steatohepatitis score (*r* = 0.74; *p* < 0.05). These results implied that MetS and histopathological behaviors were confounding factors for estimating the *μ* parameter; among various factors, the *μ* parameter exhibited the highest linear dependency on the hepatic steatosis grade.

## 4. Discussion

### 4.1. The Significance of This Study

WBV is a newly emerging alternative in treatments of hepatic steatosis. Ultrasound imaging could serve as a modality for evaluating the improvement of hepatic steatosis by WBV, depending on an effective analysis of ultrasound backscattered signals returned from the liver. In this study, ultrasound HK parametric imaging was proposed to model the backscattered statistics to evaluate the effects of WBV and exercise on changes in MetS and histopathological scores during the formation of hepatic steatosis. Compared with the control group, WBV and exercise significantly reduced body weight and improved glycemic control; they also significantly slowed the onset of pathological changes. Moreover, WBV outperformed swimming in suppressing hepatic steatosis, which can be characterized by HK imaging using ultrasound backscattered signals. This is the first study to demonstrate the utility of ultrasound HK parametric imaging in the assessment of hepatic steatosis treatment of WBV.

### 4.2. Effects of WBV on Hepatic Steatosis

Studies have explained WBV’s effects in mitigating MetS. WBV induces a tonic vibration reflex, which causes additional muscle activation during exercise and rest [41], and adaptations in energy metabolism and turnover [42]. Moreover, WBV affects the signaling pathways of obesity-related hormones, including insulin [43] and leptin [44]. On the other hand, WBV mitigated hepatic steatosis more than swimming did, as supported by the current results. As stated, exercise decreases fatty acid synthesis, increases fatty acid oxidation, and prevents hepatocellular damage by reducing the release of damage-associated molecular patterns [14]. Repeated exercise can improve hepatic steatosis because of the activation of fuel oxidation pathways in the liver [45]. However, the effect of exercise on hepatic steatosis partially depends on exercise intensity. Comparatively, WBV decreases visceral adipose tissue mass and remodels body composition even when the body weight remains unchanged [46,47]. WBV may improve systemic glucose, fatty acid metabolism, and adipokines to cause adipose tissues in the liver to contribute to hepatic steatosis producing energy to, in turn, reduce ectopic fat deposition in the liver [21]. WBV also alleviates hepatic stiffness and inflammatory effects [21].

In a previous study [48], the vibration amplitude was set at 2 mm (the same as we used) but vibration frequency was set at 30 Hz (higher than the 13 Hz used in this study). The subjects exercised for 8 weeks (3 times per week; 20 min per training containing synchronously interlaced WBV and resting) and were led by a physiotherapist to ensure the correct performance of the exercise. It showed that WBV combined with a hypocaloric diet improved body composition, insulin resistance, glucose regulation, and adiponectin levels to a greater extent compared with dieting alone [48]. This implies that WBV parameters used in this study may be applicable to human subjects after fine-tuning the parameters in combination with practical exercise and physically therapeutic intervention.

### 4.3. Changes in Acoustic Microstructures in a Fat-Infiltrated Liver

As ultrasound propagates in the liver, hepatocytes and portal triads are the major diffuse and coherent scatterers, respectively [49]. A scattering medium containing a small number of randomly located scatterers (<10 scatterers per resolution cell) with or without coherent scatterers may yield a pre-Rayleigh distribution. A scattering medium containing a large number of randomly located scatterers (≥10 scatterers per resolution cell) without or with coherent scatterers may yield a Rayleigh or Rician (post-Rayleigh) distribution, respectively [50]. In practice, the presence of small blood vessels in the liver affects the backscattered statistics of the liver [51]. As a result, the statistics of backscattered envelopes measured from a normal liver tends to be the pre-Rayleigh distribution [49].

It has been shown that decreasing the ratio of the number of coherent scatterers to the number of diffuse scatterers enables the backscattered statistics to approach the Rayleigh distribution [52]. The above process is like the progress of hepatic steatosis. Macrovesicular steatosis (a single large fat droplet existing in a hepatocyte pushing the nucleus to the periphery) is the behavior that typifies hepatic steatosis. Thus, liver tissue with hepatic steatosis can be modeled as a normal scattering medium (i.e., the liver parenchyma) embedded with fat-infiltrated hepatocytes [27]. In this condition, the first critical histopathological feature for grading the severity of hepatic steatosis is the increasing number of diffuse scatterers (fat droplets in hepatocytes), which tend to result in changes in ultrasound backscattered statistics toward the Rayleigh distribution [27]. The second critical indicator of hepatic steatosis is fat droplet size. Some small, underdeveloped fat droplets can still be observed in histopathological images even when the grade of hepatic steatosis is severe [53]. The severity of the fatty liver disease may be associated with various fat droplet sizes but no significant changes in the quantity of fat in the liver [54].

### 4.4. Usefulness of the HK Parameter in Characterizing Hepatic Steatosis

The Nakagami and HK distributions are two commonly used models for ultrasound backscattering analysis [23]. Because of the simplicity in estimating the Nakagami parameter, the Nakagami distribution has been the most frequently adopted method to describe the backscattered statistics. Thanks to advances in estimation methods for the HK parameters, using the Nakagami distribution as an approximation of backscattered statistics may not be preferred anymore; instead, the HK distribution is recommended in the context of quantitative ultrasound [23]. In addition to being generalized to model ultrasound backscattered statistics [27,28], the HK distribution also provides a relevant link between the properties of ultrasound backscattered echoes and physical interpretations of microstructures in a tissue because its *μ* parameter, as estimated using the envelope signals, is proportional not only to the number of scatterers in the resolution cell of the transducer but also to both the homogeneity of scattering cross-sections [55] and the size of fat droplets [27]. The aforementioned characteristics imply that the HK *μ* parameter depends on the number- and size-related pathological features of fat droplets, which determine the grade of hepatic steatosis and hepatocyte ballooning score.

### 4.5. Limitations and Future Work

This study has some limitations. First, the proposed method was validated using the animal model. Our results also indicated that although WBV alleviated the grade of steatosis, the steatosis grades did not return to grade 0 and the score of lobular inflammation increased. This may be attributed to the lack of diet control in the experimental design. A consensus on the vibration protocol of WBV, including factors such as amplitude, frequency, and duration, has yet to be reached [56] so potential side effects are possible (e.g., lobular inflammation observed in this study). Second, note that the yellow spot patterns exhibited in the HK images resulted in a worse smoothness to visualize the *μ* parameter, although the globally averaged *μ* characterized hepatic steatosis during WBV. Compared with this study using 11 MHz ultrasound, similar spot features were also revealed in previous studies using lower frequencies (3.5 MHz for abdominal examinations) [28,57]. Such an artifact-like pattern essentially reflects a relatively high degree of estimation biases in between local parameters. Ultrasound HK imaging using different estimators exhibits different spatial shapes and distributions in spot patterns [57,58]. On the other hand, according to the latest studies [59,60], the *μ* parameter of the HK distribution is not only related to the number of scatterers per resolution cell, but also to the packing factor, which is a function of the average and variance of the number of scatterers and may play a role in influencing the estimation value as well. The above issues imply that the effect of the packing factor and the estimator used for ultrasound HK parametric imaging may need further investigations for different ranges of frequency. In the future, appropriate protocols of WBV and improved HK parametric imaging techniques will be necessary to implement imaging-based evaluations on WBV treatments of hepatic steatosis.

## 5. Conclusions

This study conducted animal experiments to validate the performance of the proposed ultrasound HK parametric imaging in evaluating the effects of WBV on alleviating the progression of hepatic steatosis. The results demonstrated that WBV outperformed swimming to attenuate the histopathological progression in hepatic steatosis. Changes in the steatosis grade correlated with the *μ* parameter, indicating that the HK distribution endows ultrasound imaging with the ability to characterize the liver parenchyma during WBV, by establishing a physical link to the number, scattering cross-sections, and sizes of scatterers (i.e., fat droplets). WBV treatment in combination with an ultrasound backscattering analysis using the HK imaging of the liver may be a useful strategy in integrating the diagnosis and treatment of hepatic steatosis.

## Figures and Tables

**Table 1:** Changes in steatosis, steatohepatitis, and fibrosis in the sedentary group (the normality test failed, and thus ANOVA on ranks was applied).

Parameter	Control Group	SED for 4 Weeks	SED for 8 Weeks	SED for 16 Weeks	*p* Value
*μ* parameter	0.64, 0.75, 0.67 0.64, 0.65, 0.70	0.74, 0.56, 0.76 0.46, 0.85, 0.64	0.69, 1.11, 0.74 1.22, 2.74, 1.30	2.08, 4.15, 3.94 3.70, 1.42, 3.44	7.26 × 10^−6^
Weight (g)	17.43, 18.55, 18.22 17.44, 17.77, 18.54	27.06, 26.63, 26.76 25.96, 28.91, 25.98	37.21, 38.69, 39.52 41.18, 46.17, 40.21	42.76, 43.17, 42.3 43.25, 37.78, 43.58	4.76 × 10^−15^
Peak of glucose concentration (mg/dL)	231, 249, 237 232, 236, 244	413, 402, 429 394, 434, 413	361, 509, 481 493, 525, 514	447, 493, 463 482, 364, 497	4.35 × 10^−9^
Steatosis (score, 0–3)	0,0, 0, 1, 0, 0	1, 1, 0, 0, 0, 0	0, 0, 0, 2, 1, 0	2, 3, 3, 2, 2, 3	3.14 × 10^−6^
Lobular inflammation (score, 0–3)	1, 1, 0, 0, 0, 0	0, 0, 0, 0, 0, 0	0, 0, 0, 0, 0, 0	0, 0, 1, 0, 0, 1	0.26
Hepatocyte ballooning (score, 0–2)	1, 1, 0, 0, 0, 0	1, 1, 0, 0, 0, 0	0, 1, 0, 1, 1, 0	2, 2, 2, 2, 2, 2	4.75 × 10^−6^
Steatohepatitis (score, 0–8)	2, 2, 0, 1, 0, 0	2, 2, 0, 0, 0, 0	0, 1, 0, 3, 1, 0	4, 5, 6, 4, 4, 6	1.22 × 10^−6^
Fibrosis (stage, 0–4)	0, 0, 0, 0, 0, 0	0, 0, 0, 0, 0, 0	0, 0, 0, 0, 0, 0	0, 0, 0, 0, 0, 0	1

Steatohepatitis = the unweighted sum of the score of steatosis (score, 0–3), lobular inflammation (score, 0–3), and hepatocyte ballooning (score, 0–2), and thus ranges from 0 to 8.

**Table 2:** Changes in steatosis, steatohepatitis, and fibrosis in the exercise group (the normality test failed, and thus ANOVA on ranks was applied).

Parameter	Control Group	Exercise for 4 Weeks	Exercise for 8 Weeks	Exercise for 16 Weeks	*p* Value
*μ* parameter	0.64, 0.75, 0.67 0.64, 0.65, 0.70	0.82, 0.52, 0.85 0.57, 0.77, 0.85	2.32, 0.72, 0.59 1.62, 0.86, 1.26	2.10, 3.18, 2.45 3.28, 2.15, 1.31	1.78 × 10^−5^
Weight (g)	17.43, 18.55, 18.22 17.44, 17.77, 18.54	24.23, 23.46, 25.67 23.89, 24.06, 24.63	37.96, 36.11, 35.14 37.08, 36.14, 37.07	38.51, 39.87, 39.82 40.96, 39.23, 37.01	3.48 × 10^−20^
Peak of glucose concentration (mg/dL)	231, 249, 237 232, 236, 244	409, 396, 422 401, 413, 436	462, 335, 348, 457, 354, 355	382, 447, 399, 429, 388, 281	1.66 × 10^−6^
Steatosis (score, 0–3)	0,0, 0, 1, 0, 0	1, 0, 1, 0, 0, 0	2, 1, 0, 1, 0, 1	1, 2, 2, 3, 2, 1	0.0007
Lobular inflammation (score, 0–3)	1, 1, 0, 0, 0, 0	0, 1, 1, 0, 0, 0	0, 0, 0, 0, 0, 0	1, 0, 0, 0, 0, 0	0.42
Hepatocyte ballooning (score, 0–2)	1, 1, 0, 0, 0, 0	1, 1, 0, 0, 0, 0	2, 0, 2, 0, 0, 0	2, 2, 2, 2, 2, 1	0.0023
Steatohepatitis (score, 0–8)	2, 2, 0, 1, 0, 0	2, 2, 2, 0, 0, 0	4, 1, 2, 1, 0, 1	4, 4, 4, 5, 4, 2	0.0005
Fibrosis (stage, 0–4)	0, 0, 0, 0, 0, 0	0, 0, 0, 0, 0, 0	0, 0, 0, 0, 0, 0	0, 0, 0, 0, 0, 0	1

Steatohepatitis = the unweighted sum of the score of steatosis (score, 0–3), lobular inflammation (score, 0–3), and hepatocyte ballooning (score, 0–2), and thus ranges from 0 to 8.

**Table 3:** Changes in steatosis, steatohepatitis, and fibrosis in the whole-body vibration group (the normality test failed, and thus ANOVA on ranks was applied).

Parameter	Control Group	WBV for 4 Weeks	WBV for 8 Weeks	WBV for 16 Weeks	*p* Value
*μ* parameter	0.64, 0.75, 0.67 0.64, 0.65, 0.70	0.74, 0.61, 0.72 0.69, 0.54, 0.81	1.13, 2.37, 1.18 1.09, 0.56, 0.74	0.75, 0.72, 1.05 2.82, 0.65, 0.59	0.25
Weight (g)	17.43, 18.55, 18.22 17.44, 17.77, 18.54	26.01, 25.49, 24.45 24.63, 23.16, 26.13	31.29, 38.12, 36.21 30.37, 28.59, 31.22	40.38, 40.76, 41.19 41.32, 36.96, 38.62	6.69 × 10^−13^
Peak of glucose concentration (mg/dL)	231, 249, 237 232, 236, 244	401, 392, 397 389, 383, 413	486, 495, 471, 398, 327, 382	420, 431, 444, 463, 349, 352	4.38 × 10^−7^
Steatosis (score, 0–3)	0,0, 0, 1, 0, 0	0, 1, 0, 0, 0, 0	1, 2, 0, 0, 0,0	1, 1, 1, 2, 1, 0	0.0808
Lobular inflammation (score, 0–3)	1, 1, 0, 0, 0, 0	0, 0, 0, 0, 0, 0	0, 0, 0, 0, 0, 0	1, 1, 1, 1, 0, 2	0.0011
Hepatocyte ballooning (score, 0–2)	1, 1, 0, 0, 0, 0	0, 0, 1, 0, 0, 0	2, 1, 1, 1, 0, 0	1, 2, 2, 2, 2, 1	0.0007
Steatohepatitis (score, 0–8)	2, 2, 0, 1, 0, 0	0, 1, 0, 1, 0, 0	3, 3, 1, 1, 0, 0	3, 4, 4, 5, 3, 3	4.07 × 10^−5^
Fibrosis (stage, 0–4)	0, 0, 0, 0, 0, 0	0, 0, 0, 0, 0, 0	0, 0, 0, 0, 0, 0	0, 0, 0, 0, 0, 0	1

Steatohepatitis = the unweighted sum of the score of steatosis (score, 0–3), lobular inflammation (score, 0–3), and hepatocyte ballooning (score, 0–2), and thus ranges from 0 to 8.

## Data Availability

The data presented in this study are available on request from the corresponding author.
